# Priority Medicines for Maternal and Child Health: A Global Survey of National Essential Medicines Lists

**DOI:** 10.1371/journal.pone.0038055

**Published:** 2012-05-31

**Authors:** Suzanne Hill, Annie Yang, Lisa Bero

**Affiliations:** 1 Department of Essential Medicines and Pharmaceutical Policies, World Health Organization, Geneva, Switzerland; 2 Stanford Hospital & Clinics, Stanford, California, United States of America; 3 Clinical Pharmacy and Health Policy, University of California San Francisco, San Francisco, California, United States of America; Nottingham University, United Kingdom

## Abstract

**Background:**

In April 2011, the World Health Organization (WHO) published a list of “priority medicines” for maternal and child health based on 1) the global burden of disease and 2) evidence of efficacy and safety. The objective of this study was to examine the occurrence of these priority medicines on national essential medicines lists.

**Methods and Findings:**

All essential medicines lists published since 1999 were selected from the WHO website collection. The most-up-to date list for each country was then selected, resulting in 89 unique country lists. Each list was evaluated for inclusion of medicines (chemical entity, concentration, and dosage form) on the Priority Medicines List. There was global variation in the listing of the Priority Medicines. The most frequently listed medicine was paracetamol, on 94% (84/89) of lists. Sodium chloride, gentamicin and oral rehydration solution were on 93% (83/89) of lists. The least frequently listed medicine was the children's antimalarial rectal artesunate, on 8% of lists (7/89); artesunate injection was on 16% (14/89) of lists. Pediatric artemisinin combination therapy, as dispersible tablets or flexible oral solid dosage form, appeared on 36% (32/89) of lists. Procaine benzylpenicillin, for treatment of pediatric pneumonia and neonatal sepsis, was on 50% (45/89) of the lists. Zinc, for treatment of diarrhoea in children, was included on only 15% (13/89) of lists. For prevention and treatment of postpartum hemorrhage in women, oxytocin was more prevalent on the lists than misoprostol; they were included on 55 (62%) and 31 (35%) of lists, respectively. Cefixime, for treatment of uncomplicated anogenital gonococcal infection in woman was on 26% (23/89) of lists. Magnesium sulfate injection for treatment of severe pre-eclampsia and eclampsia was on 50% (45/89) of the lists.

**Conclusions:**

The findings suggest that countries need to urgently amend their lists to provide all priority medicines as part of the efforts to improve maternal and child health.

## Introduction

Improving maternal and child health is a global priority. In 2011, an estimated 7.6 million children under the age of five died [1]. Almost 358,000 women – most of them in low income countries – died from complications of pregnancy and childbirth [1]. According to the UN Commission on Life-Saving Commodities for Women and Children, many of these deaths are due to conditions such as infections, haemorrhage, pneumonia, and malaria that can be prevented or treated by affordable medicines (http://www.who.int/reproductivehealth/news/un_commission/en/index.html).

In April 2011, WHO published a list of ‘priority medicines’ for maternal and child health to support countries making choices about medicines for treating women and children [2]. These medicines were selected based on 1) the global burden of disease and 2) the evidence of efficacy and safety for preventing or treating maternal, newborn, and child mortality and morbidity. The priority medicines are a subset of the WHO Model Essential Medicines List. Priority medicines for maternal health are those for management of post-partum haemorrhage, severe pre-eclampsia and eclampsia, maternal sepsis, sexually transmitted infections (chlamydia, gonococcal infections, and syphilis), preterm birth, and maternal HIV/AIDS and malaria. For children, the list includes medicines for treating pneumonia, diarrhoea, malaria, neonatal sepsis, HIV, vitamin A deficiency, and tuberculosis. Medicines for pediatric palliative care, vitamin A deficiency, and HIV/TB prophylaxis are also included, as well as medicines for neonatal care.

Data from country surveys show that access to essential medicines, particularly for children, is generally poor [3], [4], [5], [6]. Reasons for the lack of essential medicines include fragile supply systems, out-of-pocket payments which make the medicines unaffordable, and poor quality products [7], [8]. Essential medicines should be manufactured according to quality standards, licensed for use by regulatory authorities, on essential medicines lists, part of national standard treatment guidelines, procured from the supplier of a quality product, in the supply chain, and prescribed by health care professionals who know how to use them. National essential medicines lists are a first step to ensuring access to medicines as they can guide procurement, local licensing and manufacturing, and the rational use of high quality essential medicines [9]. National essential medicines lists also help to guide allocation of limited resources and can be used as an advocacy tool to promote the accessibility of essential medicines. Therefore, national essential medicines lists are an indicator of the current supply of medicines through the public sector.

In 2007, WHO found that 131 of 151 countries surveyed had an essential medicines list.

The objective of this study was to examine the inclusion of the priority medicines for maternal and child health on national essential medicines lists.

## Methods

WHO maintains a publically available collection of the national essential medicines lists that have been submitted to WHO by member states at: http://www.who.int/selection_medicines/country_lists/en/index.html. This contained lists from 101 countries at the time this study was conducted. For this study, all essential medicines lists published since 1999, 10 years prior to the Priority Medicines list, were selected from the website. The most-up-to date list for each country was then selected, resulting in 89 unique country lists for analysis. Each EML was evaluated for concordance with the medicines listed in the WHO List of Priority Medicines for Mothers and Children 2011 [10] by AY and Monique Renevier (noted in acknowledgements). Any discrepancies were adjudicated by the other authors (SH or LB). The Priority Medicines list includes 26 individual chemical entities in 41 dosage forms and strengths.

We excluded from analysis medicines for HIV for children as these were not fully specified on the Priority List. Many countries may have separate programs for supply of antiretrovirals, so these medicines are not specified as part of a national essential medicines list. We also excluded the ‘missing’ medicines on the Priority List (formulations for treatment of paediatric TB, HIV/TB co-treatment, chlorhexidine for umbilical cord care, vitamin K for haemorrhagic disease of the newborn, and caffeine citrate for apnoea) as these formulations either do not exist, or have not yet been fully commercialized. The priority medicines listed for pediatric tuberculosis allow for combined treatments of ethambutol, rifampicin, isoniazid, and pyrazinamide. The preferable fixed dose combination of TB medicines is not available. A fixed dose combination of isoniazid and co-trimoxazole is listed for pediatric HIV/TB prophylaxis, but also does not yet exist [11]. Therefore, we assessed the concordance of 28 chemical entities in 41 dosage forms and strengths (see Table 1) with national essential medicines lists.

**Table 1:** Priority Medicines for Mothers and Children.

Condition	Chemical Entity	Concentration and dosage form
Post-partum haemorrhage	Oxytocin	Injection 10 IU or 10 IU/ml
	Misoprostol	Tablet
	Sodium chloride	Injection
	Sodium lactate (Ringer's lactate) (Hartman's)	Injection
Severe pre-eclampsia and eclampsia	Magnesium sulfate	Injection 500 mg/ml or 50%
	Calcium gluconate	Injection 100 mg/ml or 10%
Maternal sepsis	Ampicillin	Injection
	Gentamicin	Injection
	Metronidazole	Injection 500 mg (or 5 mg/ml)
Sexually transmitted diseases	Azithromycin	At least one of the forms
	Cefixime	Capsule 400 mg (or tablet)
	Benzathine benzylpenicillin	Powder for injection
Pre-term birth	Betamethasone or dexamethasone	Injection
	Nifedipine	Capsules 10 mg (or tablet)
Pneumonia	Amoxicillin	Tablet (or capsule)
	Ceftriaxone	Powder for injection
	Oxygen	Medicinal gas
	Procaine benzylpenicillin	Injection
Diarrhoea	ORS (oral rehydration salts)	Sachet
	Zinc	Tablet 20 mg
Malaria	Artemisinin combination therapy: artemether+lumefantrine or artesunate+amodiaquine or dihydroartemisinin+piperaquine	Dispersible tablet or flexible oral solid dosage form
	Artesunate	Rectal
	Artesunate	Injection
Vitamin A deficiency	Vitamin A (Retinol)	Capsule
Neonatal sepsis	Ceftriaxone	Powder for injection
	Gentamicin	Injection
	Procaine benzylpenicillin	Injection
Palliative care	Morphine	At least one of the forms (granules, injection, oral liquid, variable flexible oral solid dosage form)
	Paracetamol	Variable flexible oral dosage form (liquid, suppository, tablet)

We compared specification of the drug as the International Nonproprietary Name (INN), dosage form, and strength. For medications listed in the Priority Medicines list as ‘variable oral solid dosage forms’, any dosage forms that met the definition in the WHO Model List of Essential Medicines for Children 2010 were considered to be concordant. As the ‘scored dispersible’ 20 mg zinc tablet recommended on the Priority Medicines List is not widely available, we checked for zinc 20 mg tablets. Additionally, any artemisinin combination therapy (ACT) for malaria treatment as defined by WHO Guidelines for the Treatment of Malaria [12] was considered concordant, noting, however, that ideally, ACT for children would be in the appropriate flexible oral solid dosage forms (such as dispersible tablets).

Partial matches where the listed medicines were identical in INN and form (or the form was completely interchangeable such as tablet and capsule), but had a different strength or vial size, were discussed by two coders to decide if the match was sufficient. Medicines that were listed by INN only were not considered as fully concordant with the medicines on the Priority Medicines List.

### Analysis

We calculated the percentage of countries globally and by WHO region that included each priority medicine on its national medicines list. The most common deficiencies, as well as common inappropriate dosage forms were identified.

## Results

**Figure 1:**
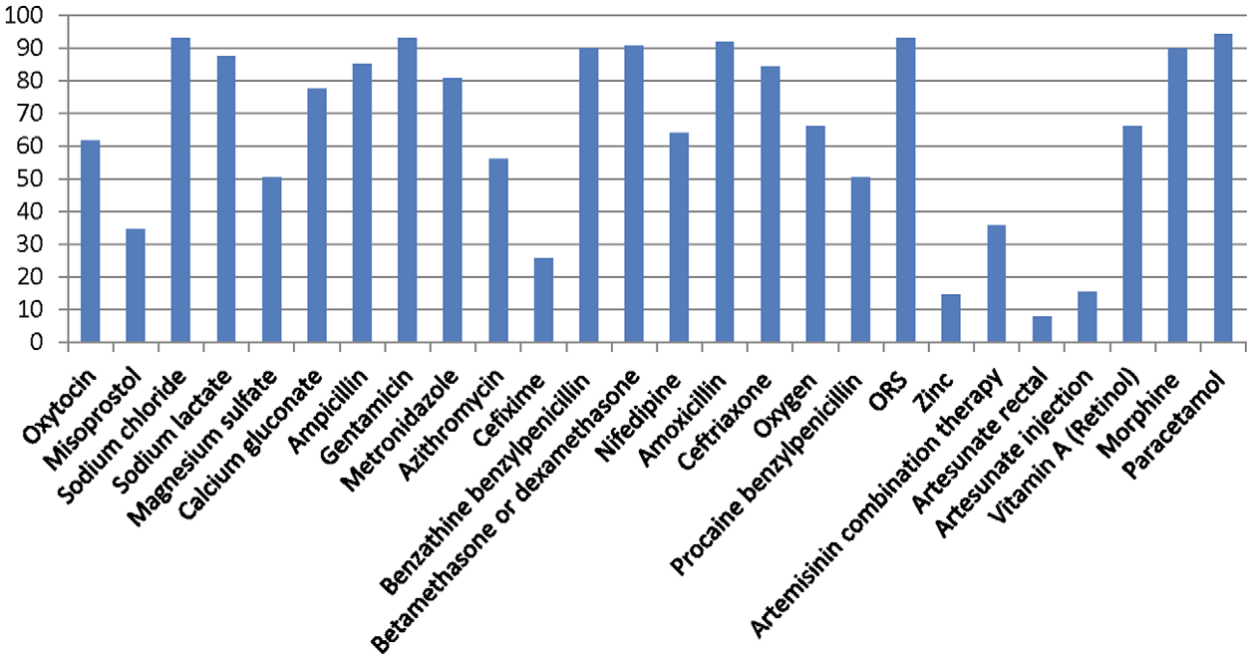
Percent of national Essential Medicines Lists (n = 89) listing each Priority Medicine for Mothers and Children. Each bar shows the percent of national Essential Medicines Lists (n = 89) listing each Priority Medicine for Mothers and Children.

As shown in Figure 1, there was considerable global variation in the listing of Priority Medicines for Mothers and Children on national essential medicines lists. Data for individual country lists can be found in the Supplemental file (Table S1). The most frequently listed medicine was paracetamol (variable flexible oral dosage form) which was on 94% (84/89) of lists. Sodium chloride, gentamicin, and oral rehydration salts (ORS) were also frequently listed and appeared on 93% (83/89) of lists.

Six medicines were listed on fewer than 50% of the lists. The least frequently listed medicine was the antimalarial rectal artesunate which was on 8% of lists (7/89); artesunate injection was on 16% (14/89) of lists. Appropriate artemisinin combination therapy, as dispersible tablets or flexible oral solid dosage form, appeared on 36% (32/89) of lists. Procaine benzylpenicillin, for treatment of pediatric pneumonia and neonatal sepsis, was on 50% (45/89) of the lists. Zinc, for treatment of diarrhoea in children, was included on only 15% (13/89) of lists.

For prevention and treatment of postpartum hemorrhage in women, oxytocin was more prevalent on the lists than misoprostol; included on 55 (62%) and 31 (35%) of lists, respectively. Cefixime, for treatment of uncomplicated anogenital gonococcal infection in woman was on 26% (23/89) of lists. Magnesium sulfate injection for treatment of severe pre-eclampsia and eclampsia was on 50% (45/89) of the lists.

Twenty-four of the national essential medicine lists were last updated prior to 2007. There was one list from 1999; all others prior to 2007 were from 2001 to 2007. The four priority medicines most recently added to the WHO Model Essential Medicines List were misoprostol, cefixime, nifedipine, and zinc, all added in 2006. The more up to date lists were more likely to contain the four medicines added in 2006 than the older lists (Table 2). One exception is nifedipine which until 2005 had been listed on the WHO Model Essential Medicines List as an antihypertensive.

**Table 2:** Listing on national essential medicines lists of four recently added to WHO Model List by date of list.

Medicine	Medicine listedN (%)	
	Lists published prior to 2007 (n = 24)	Lists published in 2007 or later (n = 65)
Misoprostol	5 (21%)	26 (40%)
Cefixime	3 (13%)	20 (31%)
Nifedipine	18 (75%)	39 (60%)
Zinc	1 (4%)	12 (18%)

**Figure 2:**
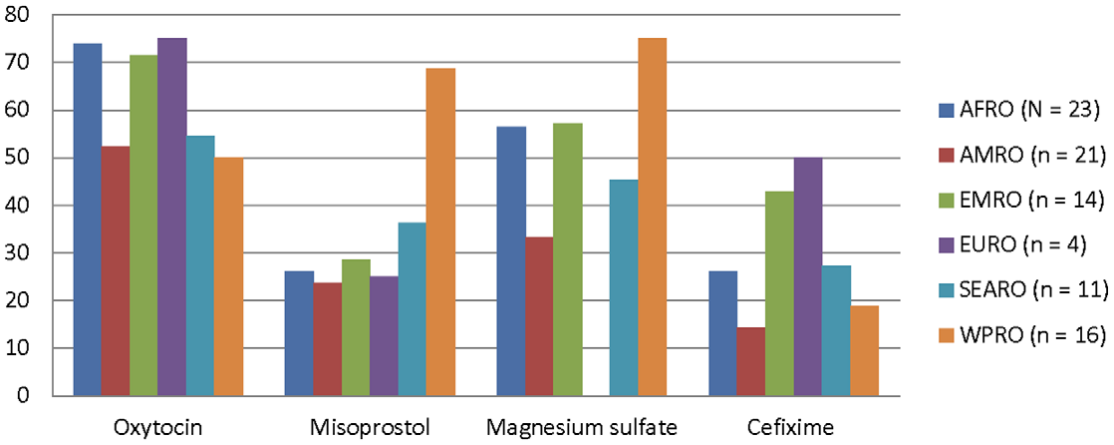
Percent of national Essential Medicines Lists listing Priority Medicines for Mothers by WHO Region. Each bar shows the percent of national Essential Medicines Lists listing Priority Medicines for Mothers by WHO Region. The abbreviations for the WHO regions are: AFRO – Africa; AMRO – the Americas; EMRO – Eastern Mediterranean; EURO – Europe; SEARO – South-East Asia; WPRO – Western Pacific.

Figure 2 shows that there is substantial regional variation in the listing of medicines for mothers. Misoprostol was more likely to be listed on country essential medicines lists in the WPRO region than any other region.

**Figure 3:**
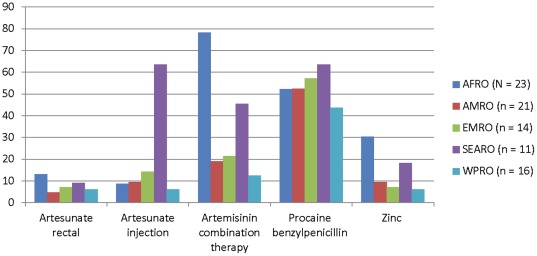
Percent of national Essential Medicines Lists listing Priority Medicines for Children by WHO Region. Each figure shows the percent of national Essential Medicines Lists listing Priority Medicines for Children by WHO Region. The 4 countries from Europe (the EURO region) were excluded from this analysis as malaria and persistent diarrhea are not prevalent in this region. The abbreviations for the WHO regions are: AFRO – Africa; AMRO – the Americas; EMRO – Eastern Mediterranean; SEARO – South-East Asia; WPRO – Western Pacific.

There was even greater global variability in the listing of selected medicines for children (Figure 3). The African continent has the highest number of malaria cases and deaths, with 174000 cases and 596,000 deaths estimated in 2010. This is followed by Southeast Asia with 28000 cases and 38000 deaths estimated in 2010 [13]. Thus, listing of the antimalarials is particularly important for the AFRO and SEARO regions. As shown in Figure 3, AFRO region countries were more likely to list artemisinin combination therapy than artesunate in either formulation, while the countries with EMLs in the SEARO region were more likely to list artemisinin combination and artesunate injection rather than artesunate rectal. The rectal form of artesunate, which is suitable for children, was not widely available in any region.

Countries with the highest incidence of malaria cases and deaths also do not uniformly list the artesunate medicines. In 2010, 15 countries (12 of which are in the AFRO region) had more than 10,000 cases of malaria per 100,000 [13]. Of these 15 countries, 7 had an essential medicines list included in this study: Burundi, Ghana, Maurtania, Senegal, Sudan, Yemen and The Solomon Islands. In the AFRO region, Burundi, Ghana, Mauritania, and Senegal listed artemisinin combination, while only Ghana listed artesunate (both rectal and injection). In the EMRO region, both Sudan and Yemen listed artemisinin combinations, Sudan listed only artesunate rectal, and Yemen listed neither preparation for artesunate. The Solomon Islands, in the WPRO region, listed all 3 antimalarials on its essential medicines list.

Procaine benzylpenicillin for pneumonia, although relatively inexpensive and widely available, was listed on only about 50% of the country lists in each region. Although the WHO recommended formulation of oral rehydration salts was found on over 90% of lists globally, zinc sulfate was listed in only 30% of countries in the AFRO region and less than 10% of countries in the AMRO, EMRO, and WPRO regions.

## Discussion

Millennium Development Goals 4 and 5 set targets for reductions in child mortality and improvements in maternal health and the UN Commission on Life-Saving Commodities for Women and Children has noted that many of these deaths can be prevented by access to basic medicines. Tracking progress towards making life-saving medicines available for children and women is needed to assess the attainment of these targets. National essential medicines lists are key policy tools for promoting the supply of priority medicines, especially through the public sector. Our findings show that essential life-saving medicines for children and mothers are not universally listed on national essential medicines lists. Key medicines that would make a potential difference in survival, such as zinc for diarrhoea in children or magnesium sulfate for eclampsia in women, are not included consistently in national documents. Many priority medicines are listed on less than 50% of the country lists analyzed. Our findings overestimate the listing of priority medicines because “missing” priority medicines, such as a fixed dose combination product for pediatric tuberculosis, were not assessed because they simply do not exist. Countries need to urgently amend their lists to provide all priority medicines as part of the efforts to achieve the targets for Millennium Development Goals 4 and 5.

WHO supports countries in the selection of essential medicines by publishing the Model Essential Medicines List. Since 2002, biannual revisions of the WHO Model Essential Medicines List have been rigorously evidence-based, and consider disease prevalence, safety and efficacy of medicines [14]. Relative cost may be evaluated as well, but no medicine is excluded from consideration because of high cost [15]. The WHO advises countries to adapt the Essential Medicines List according to their priority health care needs [14]. The process for updating the WHO Model list is transparent. Applications for deletions or additions of medicines are reviewed by a WHO Expert Committee.

The evidence provided in the applications, the committee reviews, and the summary of the discussion and decisions are publically available on the WHO website (http://www.who.int/selection_medicines/committees/expert/18/en/index.html). These publically available documents are a valuable resource for countries seeking to develop or update their essential medicines lists. Summaries and critical appraisals of the evidence reviewed by the WHO Expert committee could be used by national medicine selection committees to facilitate their own review processes. Electronic reminders, linked to evidence summaries about newly added medicines, could trigger the updating of national lists. In addition, countries could benefit from WHO guidance in how to create and operate medicines selection committees, including how to evaluate evidence and manage conflicts of interest.

A limitation of our study is that we do not describe the reasons why specific medicines are missing from specific national essential medicines lists. To do so would require an in-depth country analysis for each missing medicine because there are a variety of possible reasons, which can vary by country and medicine, for the lack of rapid policy change on national drug lists. However, our findings are consistent with a number of possible reasons for gaps in the listing of priority medicines for mothers and children on essential medicines lists. The more up to date country lists were more likely to contain the priority medicines. One exception was nifedipine which until 2005 had been listed on the WHO Model Essential Medicines List as an antihypertensive. The slight decline in listing of this drug on the national lists may be due to its removal for this cardiac indication and not reflect its addition as a tocolytic.

For some medicines, such as zinc sulfate, the desired quality dispersible product is not widely manufactured. For children under five, zinc supplementation significantly reduces the severity and duration of diarrhea [16]. Although listing of zinc on the Essential Medicines List can serve as an advocacy tool to create demand for the product, some countries may hesitate listing a product that is not widely available. The high proportion of countries listing the low osmolarity oral rehydration salts suggests that bundling or co-packaging of zinc with ORS could promote uptake when a quality zinc product is available.

For other medicines, such as artesunate, a lack of demand may be a barrier to inclusion on essential medicines lists. Artesunate injection is difficult to obtain in the AFRO region compared to SEARO. In addition, clinical practice guidelines lagged behind the Model Essential Medicines List in recommending artesunate for malaria in children. Artesunate was added to the Model List in 2000, but was not recommended as first line treatment for children until 2011 (http://www.who.int/malaria/publications/atoz/mal_treatchild_revised.pdf). The evidence supporting the use of artesunate over quinine is recent and the latest WHO guidelines have recommended either artesunate or quinine [12]. Countries may have been hesitant to list artesunate when there was little demand from practitioners.

In addition, local preferences and conditions can influence the inclusion of medicines on country essential medicines lists. For example, misoprostol was more likely to be listed on country essential medicines lists in the WPRO region than any other region. One reason for this may be that key opinion leaders in the region argued for the inclusion of misoprostol because, unlike oxytocin, it does not require refrigeration. The lack of cold storage in the region may have convinced medicines selection committees to add misoprostol.

Although listing on essential medicines lists is a first step in making priority medicines available, there can be multiple complex barriers to the use of essential medicines. Rapid country assessments of barriers to the availability and use of priority medicines are needed in order to design specific interventions to increase their appropriate use. For example, although magnesium sulfate is listed on the Essential Medicines List of Zambia, the major barrier to availability within the public health system was lack of procurement by the Ministry of Health [17]. Other barriers identified included a lack of demand by health professionals at the health center level and a lack of in-service training in the use of magnesium sulfate. Where there was demand by obstetricians, magnesium sulphate injection was being procured from the private sector by the hospital pharmacy despite not being registered and licensed for use for the treatment of severe pre-eclampsia and eclampsia by the national Medicines Regulatory Authority.

In summary, this survey of priority medicines on national essential medicines lists highlights the regional variability and poor attention to life-saving priority medicines for children and mothers. National pharmaceutical policies that include maintaining an evidence-based and up-to-date essential medicines list can be used to advocate for supply, procurement and proper use of priority medicines. Ensuring that all national essential lists include all the priority medicines would be potentially a quick win in efforts to meet the targets specified in Millennium Development Goals 4 and 5.

## Supporting Information

Table S1 Priority medicines on country essential medicines lists by country. Y = Yes, on list N = No, not on list.
(PDF)
