# Supplementary material for: Priority Medicines for Maternal and Child Health: A Global Survey of National Essential Medicines Lists
Source: PLoS One. 2012 May 31;7(5):e38055. doi: 10.1371/journal.pone.0038055 (PMC3365009; doi:10.1371/journal.pone.0038055)
Supplement: Table S1 — Priority medicines on country essential medicines lists by country. Y = Yes, on list N = No, not on list. (PDF) [file pone.0038055.s001.pdf]

Table S1: Priority Medicines on Country Essential Medicines Lists by Country

Medicine name	Medicine form	Algeria	Angola	Burundi	Cameroon	Central african republic	Chad	Congo	Democratic Congo	Eritrea	Ethiopia	Ghana	Kenya	Lesotho	Mali	Mauritania	Namibia	Nigeria	Rwanda	Senegal	South Africa
Oxytocin	**inj 10 IU or 10 IU/ml**	N	Y	Y	Y	N	Y	Y	Y	Y	Y	Y	N	Y	N	Y	Y	Y	Y	N	Y
Sodium chloride		N	Y	Y	Y	N	Y	Y	Y	Y	Y	Y	Y	Y	Y	Y	Y	Y	Y	Y	Y
Sodium lactate		N	Y	Y	Y	Y	Y	Y	Y	Y	Y	Y	Y	Y	Y	Y	Y	Y	Y	Y	Y
Magnesium sulfate	**injection 500 mg/ml or 50%**	N	N	N	N	N	Y	N	Y	Y	Y	Y	N	Y	Y	N	Y	Y	Y	N	Y
Calcium gluconate	**injection 100 mg/ml or 10%**	N	N	Y	Y	N	Y	Y	Y	Y	Y	Y	Y	Y	Y	Y	Y	N	Y	Y	Y
Ampicillin	**injection**	Y	Y	Y	Y	Y	Y	Y	Y	Y	Y	Y	N	Y	Y	Y	Y	N	Y	Y	N
Gentamicin	**injection**	Y	Y	Y	Y	Y	Y	Y	Y	Y	Y	Y	Y	Y	Y	Y	Y	Y	Y	Y	N
Metronidazole	**injection 500 mg (or 5 mg/ml)**	N	N	Y	Y	N	Y	Y	Y	Y	Y	Y	N	Y	Y	Y	Y	Y	Y	Y	N
Misoprostol	**tablet**	N	N	N	N	N	N	N	N	Y	Y	Y	N	N	N	N	N	N	N	N	Y
Azithromycin	**At least one of the forms**	Y	N	Y	N	Y	N	N	N	Y	N	Y	N	Y	Y	Y	Y	Y	Y	Y	N
Cefixime	**capsule 400 mg (interchangeable with TAB)**	Y	N	N	N	N	N	N	Y	N	N	N	N	N	Y	Y	Y	N	N	N	Y
Benzathine benzylpenicillin	**powder for inj**	Y	Y	Y	Y	Y	Y	Y	Y	Y	N	Y	Y	Y	Y	Y	Y	Y	Y	Y	Y
Betamethasone or dexamethasone	**injection**	Y	Y	Y	Y	Y	N	Y	Y	Y	Y	Y	Y	Y	Y	Y	Y	Y	Y	Y	Y
Nifedipine	**capsules 10 mg (interchangeable with TAB)**	Y	Y	N	Y	N	Y	Y	Y	Y	Y	Y	Y	Y	Y	Y	N	Y	Y	N	Y
Amoxicillin	**tablet (interchangeable with CAPS)**	Y	Y	Y	Y	N	Y	Y	Y	Y	Y	Y	Y	Y	Y	Y	Y	Y	Y	Y	Y
Ceftriaxone	**powder for inj**	N	N	Y	Y	N	Y	Y	Y	Y	Y	Y	Y	Y	Y	Y	Y	Y	Y	Y	Y
Oxygen	**medicinal gas**	N	N	Y	Y	Y	Y	Y	Y	Y	Y	Y	Y	N	Y	Y	Y	Y	Y	Y	Y
Procaine benzylpenicillin	**injection**	Y	Y	Y	N	N	N	N	N	Y	Y	N	N	Y	N	Y	Y	N	N	Y	N
ORS		Y	Y	Y	Y	N	Y	Y	Y	Y	Y	Y	Y	Y	Y	Y	Y	Y	Y	Y	Y
Zinc	**tablet 20 mg**	N	N	N	N	N	N	Y	Y	Y	N	N	N	N	Y	N	Y	N	N	N	N
Artemisinin combination therapy	**artemether+lumefantrine or artesunate+amodiaquine or dihydro + piperaquine**	N	Y	Y	N	Y	Y	Y	Y	Y	Y	Y	N	N	Y	Y	Y	N	Y	Y	Y
Artesunate	**rectal**	N	Y	N	N	Y	N	N	N	N	N	Y	N	N	N	N	N	N	N	N	N
Artesunate	**injection**	N	N	N	N	Y	N	N	N	N	N	Y	N	N	N	N	N	N	N	N	N
Vitamin A (Retinol)	**cap**	N	Y	Y	Y	N	Y	N	Y	Y	Y	Y	Y	Y	Y	Y	Y	Y	Y	Y	Y
Morphine	**At least one of the forms**	N	N	Y	Y	Y	Y	Y	Y	Y	Y	Y	Y	Y	Y	Y	Y	Y	Y	Y	Y
Paracetamol	**VARIABLE flexible SOLID Oral Dosage Form**	Y	Y	Y	Y	Y	Y	Y	Y	Y	Y	Y	Y	Y	Y	Y	Y	Y	Y	Y	Y

Table S1: Priority Medicines on Country Essential Medicines Lists by Country

Tanzania	Uganda	Zimbabwe	Argentina	Belize	Bolivia	Brazil	Chile	Colombia	Dominican Rep	Ecuador	El Salvador	Guyana	Haiti	Honduras	Jamaica	Mexico	Nicaragua	Paraguay	Peru	Suriname	Trinidad and Tobago	Uruguay	Venezuela	Afghanistan
Y	Y	N	N	Y	N	N	N	Y	Y	Y	N	N	Y	N	Y	N	N	Y	Y	Y	Y	N	Y	Y
Y	Y	Y	Y	Y	Y	Y	Y	Y	Y	Y	Y	Y	Y	Y	Y	Y	Y	Y	Y	Y	Y	Y	Y	Y
Y	Y	Y	Y	Y	Y	Y	Y	Y	Y	Y	Y	Y	Y	Y	Y	Y	Y	Y	Y	N	Y	Y	Y	Y
Y	Y	N	N	Y	N	N	N	N	N	N	Y	Y	N	N	Y	N	N	Y	Y	N	Y	N	N	Y
Y	Y	Y	N	Y	Y	Y	Y	Y	Y	Y	Y	Y	N	Y	Y	Y	Y	Y	Y	N	Y	N	Y	Y
Y	Y	Y	N	Y	Y	Y	Y	Y	Y	Y	Y	Y	Y	Y	N	Y	Y	Y	Y	Y	Y	N	Y	Y
Y	Y	Y	N	Y	Y	Y	Y	Y	Y	Y	Y	Y	Y	Y	Y	Y	Y	Y	Y	Y	Y	N	Y	Y
Y	Y	Y	N	Y	Y	Y	Y	Y	Y	Y	Y	Y	Y	Y	Y	Y	Y	N	Y	Y	Y	N	Y	Y
Y	N	Y	N	N	N	Y	N	N	N	N	Y	N	N	Y	Y	N	N	N	Y	N	N	N	N	Y
Y	Y	N	N	Y	N	N	Y	N	N	Y	N	Y	Y	Y	Y	Y	N	Y	Y	N	Y	N	Y	Y
N	N	N	N	N	N	N	Y	N	N	N	N	Y	N	N	N	N	N	Y	N	N	N	N	N	Y
Y	Y	Y	N	Y	Y	Y	Y	N	Y	Y	Y	Y	Y	Y	Y	Y	Y	Y	Y	Y	Y	N	Y	Y
Y	Y	Y	N	Y	Y	Y	Y	Y	Y	Y	Y	Y	N	Y	Y	Y	Y	Y	Y	Y	Y	N	Y	Y
Y	Y	Y	N	Y	N	Y	Y	N	Y	Y	N	Y	Y	N	N	Y	N	N	Y	N	N	N	Y	Y
Y	Y	Y	Y	Y	Y	Y	Y	y	Y	Y	Y	Y	Y	Y	Y	Y	Y	Y	Y	Y	Y	N	Y	Y
Y	Y	Y	Y	Y	Y	Y	Y	Y	Y	Y	Y	Y	N	Y	Y	Y	Y	Y	Y	N	Y	N	Y	Y
Y	Y	Y	Y	Y	Y	Y	Y	Y	Y	N	N	N	Y	N	Y	N	N	Y	Y	N	N	N	N	Y
Y	Y	Y	N	Y	Y	N	N	N	N	N	N	Y	Y	Y	Y	Y	Y	N	Y	Y	Y	N	N	Y
Y	Y	Y	Y	Y	Y	Y	Y	Y	Y	Y	Y	Y	Y	Y	Y	Y	Y	Y	Y	Y	Y	Y	Y	Y
Y	N	Y	N	N	N	N	N	N	N	N	N	N	N	N	N	N	N	Y	Y	N	N	N	N	N
Y	Y	Y	N	N	N	Y	N	N	N	Y	N	Y	N	N	N	N	N	N	N	Y	N	N	N	N
N	N	N	N	N	N	N	N	N	N	N	N	N	N	N	N	N	N	N	Y	N	N	N	N	N
N	N	N	N	N	N	Y	N	N	N	Y	N	N	N	N	N	N	N	N	N	N	N	N	N	N
Y	Y	N	N	Y	Y	Y	Y	Y	Y	N	Y	Y	Y	Y	N	N	N	Y	Y	Y	N	N	N	Y
Y	Y	Y	Y	Y	Y	Y	Y	Y	Y	Y	Y	Y	N	Y	Y	Y	Y	Y	Y	Y	Y	N	Y	Y
Y	Y	N	N	Y	Y	Y	Y	N	N	Y	Y	Y	Y	Y	Y	Y	Y	Y	Y	Y	Y	Y	Y	Y

Table S1: Priority Medicines on Country Essential Medicines Lists by Country

Djibouti	Egypt	Iran	Iraq	Jordan	Morocco	Oman	Pakistan	Somalia	Sudan	Syrian Arab Republic	Tunisia	Yemen	Armenia	Macedonia	Malta	Moldova	Bangladesh	Bhutan	DPR of Korea	India	Indonesia	Maldives	Myanmar	Nepal
Y	Y	Y	Y	N	N	Y	Y	Y	Y	N	N	Y	Y	Y	Y	N	N	N	Y	Y	Y	Y	Y	N
Y	Y	Y	Y	N	Y	Y	Y	Y	Y	Y	Y	Y	Y	N	Y	Y	Y	Y	N	Y	Y	Y	Y	Y
Y	Y	Y	Y	N	Y	Y	Y	Y	Y	Y	N	Y	Y	N	Y	N	N	N	N	Y	Y	Y	Y	Y
Y	N	Y	Y	N	N	Y	Y	Y	N	N	N	Y	N	N	N	N	N	Y	N	Y	N	Y	N	Y
Y	Y	Y	Y	N	N	Y	Y	N	Y	N	Y	Y	Y	Y	N	Y	N	Y	Y	Y	Y	N	Y	Y
Y	N	Y	N	Y	Y	Y	Y	Y	Y	N	Y	Y	Y	Y	N	Y	Y	Y	Y	Y	Y	Y	N	Y
Y	Y	Y	Y	Y	Y	Y	Y	Y	Y	N	Y	Y	Y	Y	N	Y	Y	Y	Y	Y	Y	Y	Y	Y
Y	Y	Y	Y	Y	Y	Y	Y	Y	Y	N	Y	Y	Y	Y	Y	N	N	Y	N	Y	Y	Y	Y	Y
N	N	Y	Y	N	N	N	N	N	Y	N	N	N	Y	N	N	N	Y	Y	N	N	N	Y	N	N
N	N	Y	N	Y	N	Y	Y	N	Y	N	Y	N	N	Y	Y	Y	N	N	N	Y	N	Y	Y	Y
Y	N	Y	N	Y	N	N	Y	N	Y	N	N	N	Y	Y	N	N	N	N	N	N	N	N	Y	Y
Y	Y	Y	Y	Y	Y	Y	Y	Y	Y	N	Y	Y	Y	Y	N	Y	Y	Y	Y	Y	Y	Y	Y	Y
Y	Y	Y	Y	N	Y	Y	Y	Y	Y	N	Y	Y	Y	Y	N	Y	Y	Y	Y	Y	Y	Y	Y	Y
Y	Y	y	Y	N	N	N	Y	N	Y	N	N	Y	Y	Y	Y	Y	N	N	Y	Y	Y	Y	N	Y
Y	Y	Y	Y	Y	Y	Y	Y	Y	Y	N	Y	Y	Y	N	Y	Y	N	Y	Y	Y	Y	Y	Y	Y
Y	N	Y	Y	Y	Y	Y	Y	Y	Y	N	Y	N	Y	Y	Y	Y	N	Y	N	Y	Y	N	Y	Y
Y	Y	N	N	N	N	N	Y	N	Y	Y	N	Y	Y	N	Y	Y	Y	Y	N	Y	Y	Y	Y	Y
N	Y	N	N	Y	Y	N	Y	Y	Y	N	N	Y	N	N	N	N	N	Y	Y	Y	Y	N	N	Y
Y	Y	Y	N	N	N	Y	Y	Y	Y	Y	Y	Y	Y	N	Y	Y	Y	Y	Y	Y	Y	Y	Y	Y
N	N	N	Y	N	N	N	N	N	N	N	N	N	N	N	N	N	N	N	N	N	N	N	N	Y
N	N	N	N	N	N	N	Y	N	Y	N	N	Y	N	N	N	N	Y	Y	N	N	Y	N	N	Y
N	N	N	N	N	N	N	N	N	Y	N	N	N	N	N	N	N	N	N	N	N	N	N	N	N
N	N	Y	N	N	N	N	Y	N	N	N	N	N	N	N	N	N	Y	Y	N	Y	Y	N	Y	Y
Y	Y	Y	N	N	N	N	Y	Y	Y	N	N	Y	Y	N	N	Y	Y	Y	Y	Y	Y	Y	Y	Y
Y	Y	Y	Y	N	Y	Y	Y	N	Y	Y	Y	Y	Y	Y	Y	Y	Y	Y	Y	Y	Y	Y	Y	Y
Y	Y	Y	Y	N	Y	Y	Y	Y	Y	Y	Y	Y	Y	Y	Y	Y	Y	Y	Y	Y	Y	Y	Y	Y

Table S1: Priority Medicines on Country Essential Medicines Lists by Country

Sri Lanka	Thailand	Timor-Leste	Cambodia	China	Cook Islands	Fiji	Kiribati	Malaysia	Marshall Islands	Nauru	Niue	Palau	Papua New Guinea	Philippines	Solomon Islands	Tonga	Tuvalu	Vanuatu
N	N	Y	N	N	N	Y	N	Y	Y	Y	N	Y	N	Y	N	N	Y	Y
Y	Y	Y	N	Y	Y	Y	Y	Y	Y	Y	Y	Y	Y	Y	Y	Y	Y	Y
Y	Y	Y	N	Y	Y	Y	Y	Y	N	Y	Y	Y	Y	Y	Y	Y	Y	Y
N	N	Y	N	N	Y	Y	Y	Y	Y	Y	N	Y	Y	N	Y	Y	Y	Y
Y	N	Y	N	N	Y	N	Y	Y	Y	Y	Y	Y	Y	N	Y	Y	Y	Y
Y	Y	Y	Y	Y	Y	Y	Y	Y	Y	Y	N	Y	N	Y	Y	Y	Y	Y
Y	Y	Y	N	Y	Y	Y	Y	Y	Y	Y	Y	Y	Y	Y	Y	Y	Y	Y
Y	N	Y	N	N	Y	Y	Y	N	Y	Y	Y	Y	Y	N	Y	Y	Y	Y
N	N	Y	N	N	Y	Y	Y	N	Y	Y	Y	N	Y	N	Y	Y	Y	Y
Y	N	Y	N	N	Y	N	Y	N	N	Y	N	Y	Y	N	Y	N	Y	Y
N	Y	N	Y	N	N	N	N	N	N	N	N	N	N	Y	N	N	Y	N
Y	Y	Y	N	N	Y	Y	Y	Y	Y	Y	N	Y	Y	Y	Y	Y	Y	Y
Y	Y	Y	N	Y	Y	Y	Y	Y	Y	Y	Y	Y	Y	Y	Y	Y	Y	Y
Y	N	Y	N	N	Y	Y	Y	Y	N	N	Y	Y	Y	Y	N	N	N	Y
Y	N	Y	Y	N	Y	Y	Y	Y	Y	Y	Y	Y	Y	Y	Y	Y	Y	Y
Y	Y	Y	N	Y	Y	Y	Y	N	Y	Y	Y	Y	Y	Y	Y	Y	Y	Y
Y	N	Y	N	N	Y	N	Y	N	N	Y	N	N	Y	Y	Y	Y	Y	Y
N	Y	Y	N	N	N	Y	Y	N	Y	N	Y	N	N	N	N	Y	Y	Y
Y	Y	Y	Y	Y	Y	Y	Y	Y	Y	Y	N	Y	Y	Y	Y	Y	Y	Y
Y	N	N	N	N	N	N	N	N	N	N	N	N	N	N	Y	N	N	N
Y	N	N	Y	N	N	N	N	N	N	N	N	N	N	N	Y	N	N	N
N	Y	N	N	N	N	N	N	N	N	N	N	N	N	N	Y	N	N	N
N	Y	N	N	N	N	N	N	N	N	N	N	N	N	N	Y	N	N	N
Y	Y	Y	Y	N	N	Y	Y	N	Y	N	N	N	Y	N	N	N	N	Y
Y	Y	Y	N	N	Y	Y	Y	Y	Y	Y	Y	Y	Y	N	Y	Y	Y	Y
Y	Y	Y	Y	Y	Y	Y	Y	Y	Y	Y	Y	Y	Y	Y	Y	Y	Y	Y

Table S1: Priority Medicines on Country Essential Medicines Lists by Country
